# The prospects for the SARS‐CoV‐2 pandemic in Africa

**DOI:** 10.15252/emmm.202012488

**Published:** 2020-05-05

**Authors:** Virginia Quaresima, Matteo M Naldini, Daniela M Cirillo

**Affiliations:** ^1^ Emerging Bacterial Pathogens Unit Division of Immunology, Transplantation and Infectious Diseases IRCCS San Raffaele Scientific Institute Milan Italy; ^2^ San Raffaele Telethon Institute for Gene Therapy (SR‐Tiget) IRCCS San Raffaele Scientific Institute Milan Italy

**Keywords:** Microbiology, Virology & Host Pathogen Interaction, S&S: Ethics

## Abstract

On December 31, 2019, the Chinese government officially announced the identification of a new type of coronavirus (SARS‐CoV‐2) as the etiological cause of a severe acute respiratory syndrome in Wuhan city, Hubei Province. Over the next weeks, SARS‐CoV‐2 caused a global pandemic as officially declared by the WHO on March 11, 2020, with confirmed cases and deaths in more than 166 countries. We are experiencing a worldwide phenomenon of unprecedented social and economic consequences. Since the beginning of the COVID‐19 outbreak, there have been fears that the epidemic could strongly impact weaker healthcare systems in poor‐resource settings, especially in Sub‐Saharan Africa (SSA). The 2 million Chinese nationals that live and work in Africa could potentially contribute to the spread of COVID‐19 on the continent.

## Graphical abstract

What are the prospects for the SARS‐CoV‐2 pandemic in Africa? Virginia Quaresima, Matteo Naldini and Daniela Cirillo analyse the situation in this timely commentary.

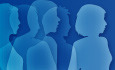

As of April 14, 2020, the World Health Organization (WHO) had reported 1,920,918 laboratory‐confirmed cases of SARS‐CoV‐2 infection and 119,686 COVID‐19 deaths worldwide. Of these, 137,895 cases and 4,964 deaths occurred in the Western Pacific Region, mainly China, and 885,103 cases with 79,783 deaths in Europe, mainly Italy (https://coronavirus.jhu.edu/data/cumulative-cases). Africa had only 15,249 confirmed cases and 816 deaths (https://africacdc.org/covid-19/). This undeniable disproportion of cases and deaths between the African region and others is striking.

## COVID‐19 is expected to hit drastically the African region

SSA countries are going through an epidemiological transition from largely communicable disease‐caused burden to an increasing predominance of the main chronic non‐communicable diseases: cardiovascular diseases, cancer, chronic respiratory diseases, and diabetes. Data from China and Western Countries confirm that these comorbidities—along with others, such as age and obesity—are risk factors for developing a severe respiratory syndrome from SARS‐CoV‐2 (Zhou *et al*, 2020). Furthermore, the number of undernourished people in SSA rose from 181 million in 2010 to almost 222 million in 2016 while the percentage of overweight or obese adults increased from 28.4% in 2000 to 41.7% in 2016 (https://www.afro.who.int/sites/default/files/2019-08/AFR-RC69-7%20Strategic%20Plan%20to%20reduce%20the%20double%20burden%20of%20malnutrition.pdf). SARS‐CoV‐2 infection could therefore have a tremendous impact on African populations facing the double burden of malnutrition and obesity along with diabetes, cardiovascular diseases and respiratory diseases.

Furthermore, the affliction of communicable diseases in Africa remains very high and affects the most vulnerable and disadvantaged communities. In 2018, the African Region counted 25.7 million people living with the human immunodeficiency virus (HIV) and almost one‐third of new HIV cases worldwide (https://www.afro.who.int/health-topics/hivaids). In addition, 25% of global tuberculosis (TB) deaths occurred in Africa, where multidrug‐resistant TB is an emerging threat (https://www.afro.who.int/health-topics/tuberculosis-tb).

Compared with all other regions in the world, Africa fights against the greatest infectious disease burden with the weakest public health infrastructure, which is often concentrated at major urban settings leaving the countryside unserved. Most SSA countries suffer a chronic shortage of healthcare workers and hospitals and clinics are already operating at maximum capacity, leaving little margin for treatment and management of COVID‐19 patients. Moreover, many countries rely on external donor funding for specific health programs, such as against malaria, HIV, or TB, and such sources may become unreliable as the current health and economic crisis affects Western countries.

In many low‐ and middle‐income countries (LMICs), a Central Medical Store stocks and delivers necessary items to the lower administrative levels—regions and districts—without local storage points for stocking emergency supplies. Such a system may not be able to provide high‐quality products to all geographic locations during peak requests owing to stockouts, failure to properly forecast future demands, and delayed responses. Importantly, as Western countries aggressively acquire scarcely available test kits and protective equipment, Africa might face competition with first‐world countries to assure its own resources.

In a fight against a rapidly spreading disease, time is of the essence. Past experience from other communicable diseases in SSA has highlighted frequent delays between the acquisition and transportation of diagnostic samples to laboratories and the notification of test results back to health facilities which has a negative impact on treatment outcomes. A weak sample handling and referral system could represent another major barrier for establishing an efficient SARS‐CoV‐2 contact tracing network.

Given all these factors, the African region can be considered a high‐risk priority zone for proactive surveillance, detection, and containment of SARS‐CoV‐2, with central coordination of all interventions to reduce the burden on healthcare services.

## Is Africa getting ready for the fight?

On February 5, 2020, even before the first confirmed COVID‐19 case on the continent, the Africa CDC established the Africa Task Force for Novel Coronavirus (AFCOR) to oversee preparedness and responses. On February 20, an emergency meeting of the ministers of health of the 55 African member states was held in Addis Ababa, Ethiopia, to commit all states to act fast and collectively and to develop and implement a coordinated continent‐wide strategy (see Table 1 for a summary of key initiatives by AFCOR and Partners for handling the outbreak) (https://africacdc.org/download/outbreak-brief-11-covid-19-pandemic-31-march-2020/).

**Table 1:** Main AFCOR activities (https://africacdc.org/download/outbreak-brief-11-covid-19-pandemic-31-march-2020/)

	Main AFCOR proposed/performed activities
Surveillance/points‐of‐entry screening	• Training of trainers’ events in 18 African countries to enhance surveillance at points‐of‐entry. • Training of the first 7 out of 20 countries in event‐based surveillance by the US CDC. • Airport staff training in South and East African regions. More than 200 participants from 10 member states (Malawi, Uganda, Zimbabwe, Lesotho, Botswana, Rwanda, Tanzania, Eswatini, Ethiopia, and Namibia) were trained.
Infection prevention and control	• Implementation of social distancing strategies. • Lockdown and closing of non‐essential activities and services.
Clinical management of severe infections	• Africa CDC has established a continent‐wide network of clinicians who had been involved in webinar that included about 300 clinicians. • Online portal with training materials such as courses, online case studies, and social media vignettes to support evidence‐based care of COVID‐19 patients.
Lab diagnostics and subtyping	• Availability of detection kits, swabs face masks, and protective clothing to 54 Member States. • Contact with main manufacturers for alternative and simple testing strategies. • The African Society for Laboratory Medicine has arranged online training program for more than 40 laboratories (March 25 and 30, 2020). • Shortage of viral transport media, swabs, and extraction kits is the next challenge to be faced for expanding testing.
Risk communication and community engagement	• From early January, CDC Africa created a dedicated webpage for COVID‐19 with weekly outbreak reports and useful educational materials for infection prevention and control. • Risk communication trainings for public health officers of 27 countries to manage outgoing information flow for COVID‐19. • Risk communication WhatsApp group for the communication officers that were trained to share information and minimize the circulation of rumors. • WhatsApp group with over 100 journalists to share updates about the outbreak in Africa and how Africa CDC and the countries are responding. • Weekly podcast on the outbreak and production of documentaries providing key educational materials for the public. • Co‐Creation Hub (CcHUB) online platform and Africa CDC support innovative communication projects on COVID‐19 to catalyze citizens actions and fight disinformation.

Strategies to slow or stop the spread of any infectious disease, including the current COVID‐19 pandemic, follow the imperative triple “T” motto: Trace, Test, and Treat. Most, though not all, affected countries have started testing everyone with symptoms, tracking their contacts and isolating them. Consequently, countries all over the world are now competing for SARS‐CoV‐2 test kits and respiratory support devices. The USA and the UK are already securing reagents for their national needs and US diagnostic companies have been banned from exporting SARS‐CoV‐2 tests, which leaves the rest of the world, and especially Africa, with fewer suppliers and limited possibilities for manufacturing.

On the positive side, the WHO African regional office launched, back in 2009, the Stepwise Laboratory Quality Improvement Process Towards Accreditation (SLIPTA) program with the aim to strengthen laboratories’ compliance with international standards through training and mentoring. This has greatly expanded the number of professional laboratory scientists and managers.

During the 2014 Ebola outbreak, fast roll‐out of molecular diagnostics helped to contain the epidemic. Similarly, SSA countries could take advantage of the large number of existing GeneXpert^®^ tools, located at both central and peripheral laboratories. GeneXpert^®^ is a multi‐disease diagnostic platform used initially to test TB and later adapted for HIV and Ebola. The GxAlert, a web‐based data management system associated to GeneXpert^®^, allows automatic transfer of test results from the diagnostic device to the GxAlert web server. This avoids delays in notifications and helps to monitor testing in the whole country, two key aspects for successful containment of an emerging infectious outbreak. As of March 21, thanks to an emergency approval by the US FDA, GeneXpert Xpress SARS‐CoV‐2 cartridges are available on the market. If properly rolled out, such technology will allow to decentralize SARS‐Cov‐2 testing and provide results in less than 45 min with minimal staff training. On the other hand, the implementation of GeneXpert presents major challenges, particularly related to cost and infrastructure. The price of GeneXpert equipment and cartridges had been an undeniable barrier for scaling up this technology in many LMICs and could be a major problem during the current pandemic.

It is also crucial that other major communicable diseases, which are highly endemic in SSA and require yearly extraordinary efforts from each country's health system, are not neglected or disregarded during the current COVID‐19 epidemic: the use of GeneXpert^®^ platforms for COVID‐19 testing may impact the diagnosis of TB and HIV. Strategies involving next‐generation “health‐tech” and digital solutions, such as those proposed by the Stop TB partnership to diagnose pulmonary infections and monitor TB treatment adherence (fundamental aspect of TB therapy and spread control), will need to be implemented to mitigate disruption in routine health services while battling against SARS‐CoV‐2.

## Limitations to the implementation of preventive measures during the COVID‐19 outbreak

Tough measures have been adopted by African countries for mitigating the spread of the virus, such as full lockdowns, shutting down airports, and imposing travel restrictions. South Africa, which has registered the highest number of SARS‐CoV‐2 cases (2,272 cases as of April 14) within SSA, has officially announced an unprecedented lockdown of 21 days which started on March 26. On March 17, Nigeria restricted entry for travelers from countries highly affected by COVID‐19 and, on March 23, shut down its two main international airports in the cities of Lagos and Abuja. Ethiopia, Ghana, Zambia, Congo (Republic), Rwanda, and many others have also closed their borders and suspended air travel.

In order to mitigate intra‐community transmissions, many countries have implemented social distancing measures, which pose a particular challenge for SSA countries. Households are generally shared by many families. About 43% of the continent's population lives in overcrowded settings, with up to 77% in major cities such as Lagos (Nigeria) where 8–12 people may live in dwellings with only two rooms. Studies have associated overcrowded housing with increased risk of respiratory diseases, such as TB, influenza, meningococcal disease, pneumonia, and other acute respiratory infections; as such, the crowded housing conditions provide an ideal scenario for the rapid spread of SARS‐CoV‐2.

Furthermore, lack of hygiene poses another challenge for social distancing measures. According to the 2014 report from the Ghana Statistical Service (GSS) (https://www.statsghana.gov.gh/), 60% of the population in the Ashanti region in the center of Ghana lives in urban areas with no indoor toilets, potable water, electricity, or waste disposal facilities. About 41% of the population has access only to public toilets shared by many households. In Ghana, as in most parts of SSA, water is commonly collected from standpipes, boreholes, and protected wells; charcoal is still the primary source for cooking, and cooking is generally conducted in open spaces, among the household inhabitants. Furthermore, crowded street markets are the main if not exclusive source for essential goods. It is difficult to foresee how social distancing measures and increased hygiene practices can be achieved while assuring the minimal means of sustainment to the community.

Indeed, many concerns have risen on how families will financially support themselves during the imposed lockdowns as most of the country's population relies on daily income for sustenance. BBC Africa reports that many Nigerians live hand‐to‐mouth, with daily budgets of less than US$1 and no possibility of stocking up on food or other essentials goods. Following the western model, urgent financial support and welfare measures are needed in LMICs to avoid widespread bankruptcies and guarantee access to food, shelter, and basic needs. Africa may not be able to provide on its own such measures, and local populations may worry more about hunger than COVID‐19.

## Could SARS‐CoV‐2 hit mildly SSA?

The relatively low number of SARS‐CoV‐2 confirmed cases in Africa so far has been explained as the effect of poor notifications and low testing rates. However, this hypothesis has yet to be investigated and documented.

During the early stage of the outbreak, a research team from the University of London analyzed flight data for passengers traveling from the four major Chinese cities of Wuhan, Beijing, Shanghai, and Guangzhou since October 2014 to predict the development of the infection. Their model of 388,287 passengers traveling to 1,297 airports, estimated that the risk of transmission to Africa and South America was relatively low. Conversely, Asian and European countries showed a high risk of transmission (Haider *et al*, 2020). In line with this prediction, Europe and North America are now the most affected regions by COVID‐19 in the world, while SSA lags far behind.

A second strong advantage is Africa's very young population as age is among the top risk factors for developing a severe acute respiratory syndrome necessitating intensive care. In the Chinese data, COVID‐19 crude fatality rate (CFR) for people older than 80 years increases up to 21.9%. While in 2019 the number of people aged 65 years or older in Europe and North America were 200.9 million (18% of the population), SSA has a very young population in comparison, with a median age of 19.7 years and only 31.9 million people or 3% of the population aged 65 years or older (https://www.un.org/en/development/desa/population/publications/pdf/ageing/WorldPopulationAgeing2019-Highlights.pdf). We can thus hope for a lower percentage of severe SARS‐CoV‐2 cases necessitating intensive treatment that may not uniformly be available across SSA.

Climate can also act as a possible mitigating factor. Human coronavirus, such as SARS‐CoV and SARS‐CoV‐2, is a RNA virus belonging to the *Coronaviridae* family, which are characterized by, but not limited to, winter seasonality with peaks of activity between December and April in the Northern hemisphere. Initial reports have also speculated that heat and humidity might hamper the spread of SARS‐CoV‐2. Yet, additional epidemiologic data on geographic and environmental factors are needed for a better understanding of SARS‐CoV‐2 circulation in tropical climates.

Genetic factors could play a protective role too. The main cell receptor for SARS‐CoV‐2 is angiotensin I converting enzyme 2 (ACE2), a homolog of ACE (Hoffmann *et al*, 2020). ACE2 is involved in the renin–angiotensin–aldosterone system (RAAS) that regulates blood pressure and is expressed in epithelial cells of the lung and other organs. ACE‐inhibitors, commonly prescribed blood‐pressure drugs, are less effective in African Americans compared with Caucasians, which has been linked to genetic predisposition of African Americans to higher baseline RAAS activity with increased susceptibility to salt retention rather than to the ACE protein itself (Spence & Rayner, 2018). There are limited data associating genetic background to SARS‐CoV‐2 pathogenicity and no evidence so far of ACE2 polymorphisms that confer resistance to viral infection. Recent single‐cell RNA‐sequencing expression data (preprint: Zhao *et al*, 2020), although limited in the numbers of donors analyzed, noticed that lung tissue from an Asian male had significantly higher ACE2‐expressing cell content than Caucasian or African American donors. Such findings might suggest different susceptibility to infection or to the severe forms of COVID‐19 based on ethnic background but must be further validated in appropriate studies.

In SSA, the “germ theory of infectious disease” is not unquestionably accepted by all cultures and illnesses are often ascribed to supernatural causes. Social scientists therefore played a decisive role during the 2014 Ebola outbreak by engaging with local communities to intervene on customary traditions which could further enhance viral spread, such as safe burial of Ebola victims and avoiding traditional washing of the corpse by the family. Thus, it is of vital importance to acknowledge and understand local customs and cultural beliefs so as to apply and adapt infection control policies. From such past experience, SSA has learnt positive lessons and pre‐existing isolation facilities which may aid in its future battle against SARS‐CoV‐2.

## African migrants in Italy

Northern Italian regions have been the area most hit by COVID‐19 so far and also host the nation's highest rate of foreign citizens in the country. In 2019, 5.2 million foreigners were living in Italy and 22.5% of them just in Lombardy; 25% of them were African migrants; 67% from North Africa; and 33% from SSA (https://www.tuttitalia.it/lombardia/statistiche/cittadini-stranieri-2019/). Preliminary and anecdotal data gathered from Italian hospitals have revealed a low percentage of COVID‐19 severe patients from SSA. This relatively low number could be caused by younger asymptomatic carriers, poor self‐reporting of mildly symptomatic patients, and/or from limited access to the healthcare system. The COVID‐19 cohort of the Lombardy region, and in general of Italy, could give new insights into the impact of SARS‐CoV‐2 on different populations with diverse genetic backgrounds, including people from SSA.

The comparison of outbreak evolution in two northern Italy regions, Lombardy and Veneto, applying different infection control strategies, shows that aggressive testing alongside shifting from a nosocomial centered model of care to a decentralized system in local communities reduces the number of deaths and the number of infections among healthcare workers. Learning from the Italian lesson, testing should be promoted and combined with rigorous contact tracing and isolation, along with reliable communication systems for collecting and sharing information. In SSA, community‐based approaches should be enforced by promoting out‐reach strategies to provide care at the local level.

In conclusion, SSA could be at high risk for severe COVID‐19 spread and fatality owing to the high burden of both infectious diseases and NCDs. Nevertheless, the African region has set in motion control and prevention strategies, such as the African Coronavirus Taskforce, and may exploit existing GeneXpert network for SARS‐CoV‐2 diagnosis and case tracing. National healthcare programs and international organizations have readily acted to assure the continuation of treatment and assistance of patients with TB and HIV. Hopefully soon, international alliances could further support SSA countries in the fight against other major communicable diseases, which imperatively should not be disregarded despite the COVID‐19 epidemic. Last, but not least, the SSA population age distribution greatly favors a milder affection by SARS‐CoV‐2.

Worldwide COVID‐19 data collected so far lack stratification by ethnicity which could assist countries and policy makers by helping predict the development of the pandemic in the SSA region and shed light on SARS‐CoV‐2 pathogenic processes across different genetic backgrounds.
